# Serotonin Transporter Genotype Modulates the Gut Microbiota Composition in Young Rats, an Effect Augmented by Early Life Stress

**DOI:** 10.3389/fncel.2017.00222

**Published:** 2017-08-03

**Authors:** Sahar El Aidy, Anouschka S. Ramsteijn, Francisco Dini-Andreote, Roel van Eijk, Danielle J. Houwing, Joana F. Salles, Jocelien D. A. Olivier

**Affiliations:** ^1^Microbial Physiology, Groningen Biomolecular Sciences and Biotechnology Institute, University of Groningen Groningen, Netherlands; ^2^Neurobiology, Groningen Institute for Evolutionary Life Sciences, University of Groningen Groningen, Netherlands; ^3^Microbial Ecology Cluster, Genomics Research in Ecology and Evolution in Nature, Groningen Institute for Evolutionary Life Sciences, University of Groningen Groningen, Netherlands

**Keywords:** serotonin transporter, intestinal bacteria, maternal separation, wild type, heterozygous, knockout rats

## Abstract

The neurotransmitter serotonin (5-HT) plays a vital regulatory role in both the brain and gut. 5-HT is crucial for regulating mood in the brain as well as gastrointestinal motility and secretion peripherally. Alterations in 5-HT transmission have been linked to pathological symptoms in both intestinal and psychiatric disorders and selective 5-HT transporter (5-HTT) inhibitors, affecting the 5-HT system by blocking the 5-HT transporter (5-HTT) have been successfully used to treat CNS- and intestinal disorders. Humans that carry the short allele of the 5-HTT-linked polymorphic region (5-HTTLPR) are more vulnerable to adverse environmental stressors, in particular early life stress. Although, early life stress has been shown to alter the composition of the gut microbiota, it is not known whether a lower 5-HTT expression is also associated with an altered microbiome composition. To investigate this, male and female wild type (5-HTT^+/+^), heterozygous (5-HTT^+/-^), and knockout (5-HTT^-/-^) 5-HT transporter rats were maternally separated for 6 h a day from postnatal day 2 till 15. On postnatal day 21, fecal samples were collected and the impact of 5-HTT genotype and maternal separation (MS) on the microbiome was analyzed using high-throughput sequencing of the bacterial 16S rRNA gene. MS showed a shift in the ratio between the two main bacterial phyla characterized by a decrease in *Bacteroidetes* and an increase in Firmicutes. Interestingly, the 5-HTT genotype caused a greater microbal dysbiosis (microbial imbalance) compared with MS. A significant difference in microbiota composition was found segregating 5-HTT^-/-^ apart from 5-HTT^+/-^ and 5-HTT^+/+^ rats. Moreover, exposure of rats with 5-HTT diminished expression to MS swayed the balance of their microbiota away from homeostasis to ‘inflammatory’ type microbiota characterized by higher abundance of members of the gut microbiome including *Desulfovibrio*, *Mucispirillum*, and *Fusobacterium*, all of which are previously reported to be associated with a state of intestinal inflammation, including inflammation associated with MS and brain disorders like multiple depressive disorders. Overall, our data show for the first time that altered expression of 5-HTT induces disruptions in male and female rat gut microbes and these 5-HTT genotype-related disruptions are augmented when combined with early life stress.

## Introduction

Serotonin is a key neurotrophic factor and critical in fetal brain development (Gaspar et al., 2003). Disturbances in the serotonergic system increase the susceptibility to anxiety and depression (Olivier et al., 2014), and early life stress seems to augment this risk (Carola et al., 2008). Besides its role in the brain, 5-HT has diverse roles in the gastrointestinal tract, ranging from modulation of electrolyte absorption, maintenance of fluid homeostasis, alterations in gastrointestinal motility, and regulation of gut permeability (Coates et al., 2006; Haub et al., 2010; Gill et al., 2013). High 5-HT levels are implicated in the pathophysiology of carcinoid syndrome, dumping syndrome, inflammation, or enteric infections (Spiller, 2008). Since 5-HT mediates its actions via various receptor subtypes, it is critical to maintain optimal extracellular availability of 5-HT in the gut to facilitate its physiological actions and prevent alterations in the sensitivity of the receptor. In this regard, the intestinal 5-HT transporter (5-HTT) plays a key role in clearance of 5-HT (Martel, 2006; Gill et al., 2008). Decreased expression of 5-HTT and consequent high 5-HT levels have been implicated in various inflammatory and diarrheal disorders. For example, 5-HTT expression is decreased in ulcerative colitis patients and in several experimental models of colitis (Coates et al., 2004; Faure et al., 2010). Similarly, targeted deletion of the 5-HTT in mice results in an abnormal pattern of motility, and exacerbation of inflammatory responses (Chen et al., 2001; Bischoff et al., 2009). Thus, appropriate 5-HTT regulation is critical for maintenance of 5-HT homeostasis in the gut.

An increasing body of evidence suggests that the gut microbiota is part of a complex bidirectional communication network between the central nervous system and gastrointestinal tract (Carabotti et al., 2015). This interaction is also known as the brain-gut-microbiota signaling system, in which the intestinal microbial community plays a key role in the regulation of stress and early life programming of the neuro-immune system (Dinan and Cryan, 2012).

Recently, it has been shown that the production of 5-HT in the gut is dependent on the gut microbiota. Especially spore-forming bacteria are important modulators of host colonic and blood 5-HT. Particular microbial metabolites, namely short chain fatty acids have been shown to be elevated by spore-forming microbiota and subsequently promoted 5-HT levels in endochromaffin cells in the epithelia, regardless of the 5-HTT (Yano et al., 2015). In addition, it has become clear that 5-HT not only plays an important role in gut functioning (Gershon and Tack, 2007), but also that brain-related 5-HT functions and symptoms are mirrored in the gut microbiome. For instance, in depressed patients with low levels of 5-HT, an enrichment of *Alistipes* (a *Bacteroidetes* species) was found in the gut (Naseribafrouei et al., 2014). *Alistipes* influences tryptophan, the precursor of 5-HT and consequently the availability of 5-HT (Song et al., 2006) thereby altering the gut 5-HTergic system. Similarly, the gut microbiome has also been shown to influence central 5-HT levels. For example, *Bifidobacterium infantis* has been suggested to increase plasma tryptophan levels, which may influence central 5-HT transmission (Desbonnet et al., 2010). The gut microbiota has the ability to control 5-HT levels and the production of neuroactive metabolites (O’Mahony et al., 2015).

Despite these reports suggesting an influence of disturbed levels of 5-HT on the gut microbiota and acknowledging the importance of 5-HTT as a novel target for gastrointestinal disorders, it is not known whether dysregulation of 5-HTT is associated with alterations in the composition of gut microbiota.

In rats, maternal separation (MS) in the early postnatal period has often been used as a stressor and can produce lasting effects in a.o. emotionality and responsivity to stressors later in life (Holmes et al., 2005). A very large majority of MS protocols vary from 3 to 6 h, with some exceptional longer, 12 h, protocols. Although prolonged MS (up to 6 h) increases the sensitivity to stress, brief handling (15 min) seems to attenuate this effect (for review see, Holmes et al., 2005; Cannizzaro et al., 2007). The 5-HTT genotype seems to influence the effect of maternal care, but is only manifest in individuals exposed to prolonged or repeated stress (Caspi et al., 2002; Huang et al., 2004).

In order to determine whether repeated early life stress combined with altered 5-HTT expression influences the composition of the gut microbiota in young rats, we maternally separated 5-HTT^+/+^, 5-HTT^+/-^, and 5-HTT^-/-^ for 6 h/day rats from postnatal day (PND) 2 till PND 15 and sampled their feces at PND 21. This time point was chosen because it captures an important moment in development as the offspring are transitioning from mother’s milk to solid food, which likely constitutes an important milestone on the way to adulthood in terms of the maturation of their metabolism and the development of the adult gut microbiome. We hypothesize that animals that underwent early life stress will display microbial dysbiosis as previously shown (O’Mahony et al., 2010; De Palma et al., 2015; Pusceddu et al., 2015). In addition we expect microbial dysbiosis to be more pronounced in rats lacking the 5-HTT and heterozygous 5-HTT knockout animals, although to a lesser extent than homozygous knockouts. Finally, as females are more vulnerable to develop depression than males, we expect a sex difference in the microbiota composition. Data about the PND 21 microbiome can generate hypotheses about the possible long-term effects of both early life stress as well as 5-HTT genotype and thereby give us important future study design.

## Materials and Methods

### Animals

Serotonin transporter knockout rats (*Slc6a4*^1Hubr^, 5-HTT^-/-^) were bred in our facility by crossing 5-HTT^+/-^ females with 5-HTT^+/-^ male rats, resulting in offspring of the three genotypes (5-HTT^+/+^, 5-HTT^+/-^, and 5-HTT^-/-^). Pregnant dams were housed in standard Macrolon type III cages containing wood chip bedding material and Enviro-dri^®^as enrichment. Rats had *ad libitum* access to water and food (RMH-B, AB Diets; Woerden, the Netherlands) in a temperature (21 ± 1°C) and humidity-controlled room (45–60% relative humidity), with a 12 h light/dark cycle (lights off at 10:00 a.m.). The females were inspected daily for delivery of pups at 5:00 p.m., and day of birth was designated as postnatal day 0. All experimental procedures were approved by the Groningen University Committee of Animal experiments. For a detailed timeline of the experiment see Supplementary Figure 1.

### Maternal Separation

Litters were randomly allocated to one of two rearing conditions (from PND 2 till PND 15): maternal separation for 360 min (MS) or control handling for 15 min (CTR). Upon reunion of mothers and pups, mothers rearrange their nests and provide maternal care to the pups. Short handling (15 min), and thus the maternal care provided upon reunion, has shown to improve cognitive performance and reduce emotionality and stress responses (Pryce and Feldon, 2003; Cannizzaro et al., 2005; Raineki et al., 2014; reviewed in Pryce and Feldon, 2003). Short handled (15 min) rats are different from rats raised in standard animal facility rearing conditions. We therefore chose to use the short handling (15 min) protocol to control for the effect of maternal care upon reunion with the mothers. MS was started daily between 8:30 and 9:30 a.m., and was performed as follows: the pups were removed from the home cage and placed as a whole litter into a smaller cage (macrolon type IL) with only sawdust bedding after which they were transferred to an adjacent room. Only rats participating in this MS protocol from this study were present in this room. The temperature of the separated litters was set to maintain 32 ± 1°C from PND2 to PND8 and 28 ± 1°C from PND9 to PND15 by placing the cages on a heat mat. Control animals were maternally separated and handled for 15 min. Control pups stayed in the same room as their respective mother. At the end of the separation period, litters were returned to their home cage by placing them in the nest and covering them with some home cage bedding material. Home cages were refreshed at PND7 and PND14. At PND21, ear punches were taken of the pups for identification and genotyping and pups were weaned.

### Fecal Sample Collection

Fecal pellets were collected from PND21 old male and female 5-HTT^+/+^, 5-HTT^+/-^, and 5-HTT^-/-^ rats (*n* = 8 per group). All samples were immediately frozen in liquid nitrogen and stored at -80°C until further processing.

### Genotyping

Ear punches were lysed overnight in 400 μL lysis buffer, containing 100 mM Tris (pH 8.5), 200 mM of NaCl, 0.2% of sodium dodecyl sulfate (SDS), 5 mM of ethylene diamine tetraacetic acid (EDTA), and 100 μg/ml of freshly added Proteinase K at 55°C. The next day, proteinase K activity was ended by 10 min incubation at 80°C. Samples were cooled and shortly centrifuged to collect condensate. DNA was precipitated by adding 400 μL isopropanol, mixing by invertion, followed by centrifuging for 10 min at 14.000 *g*. The supernatant was removed by gently inverting the tube and the pellets were washed with 300 μL 70% ethanol, centrifuged for 5 min at 14.000 *g*. The supernatant was carefully discarded and the pellets were set to air-dry. The pellets were eluted in 100 μL TE-buffer by incubating 10 min at 70°C followed by vortex. For genotyping purposes the following primers and probes were used: Forward primer: 5′-GCACGAACTCCTGGAACACT, Reverse primer: 5′-AGCGTCCAGGTGATGTTGTC, 5-HTT wild type probe: 6FAM-AGTTGGTGCAGTTGC-MGBNFQ 5-HTT knockout probe: VIC-AGTAGTTGGTTCAGTTGC-MGBNFQ (solved in 20x primer solution, Life Technologies, the Netherlands).

Genotyping was performed using Applied Biosystem 7500 fast (Life Technologies, the Netherlands). The total reaction was 25 μL containing 12.5 μL Taqman Universal Mastermix II, no UNG (cat# 4440047, Life Technologies, the Netherlands); 1.25 μL 20x primer solution; 10.25 μL sterile H_2_O and 1 μL DNA sample. The thermal cycling for genotyping was as follows: 95°C 10 min +40× (92°C 15 s + 60°C 1 min). Genotypes were manually inspected by comparison with the parallel runs of positive controls.

### Microbiota Composition Analysis

DNA was extracted from fecal samples using the PowerFecal DNA Isolation Kit (Mo Bio Laboratories, Inc., Carlsbad, CA, United States) following the manufacturer’s protocol. DNA concentration and purity (260/280 and 260/230 ratios) was quantified using NanoDrop 2000c (Thermo Scientific^TM^) and samples were thereafter stored at -20°C until further use.

In triplet a total of 10 ng μL^-1^ of extracted DNA per sample was used for PCR-amplification of a 400 bp fragment of the bacterial 16S rRNA gene using a primer set with a sample specific barcode sequence (see **Table 1** for details). The following 25 μL master mix was used: 200 nM dNTPs mix, 1X HF-Buffer, 500 nM forward and reverse primers, 0.5 U Phusion DNA polymerase (Thermoscientific^TM^). To ensure the specificity of the reaction, the following PCR conditions were set: initial denaturation step at 98°C for 30 s, followed by 30 cycles of 98°C for 10 s, 70°C for 30 s, 72°C for 30 s; with a final step of 72°C for 7 min. After amplification the triplet PCR mixtures were pooled and loaded on a 2% agarose gel stained with SYBR-safe (Thermoscientific^TM^) and the 400 bp fragments were excised. DNAs were extracted from the excised agarose parts using the Qiaex II gel extraction kit (Qiagen, the Netherlands) following the manufacturer’s protocol. Purified fragments were eluted in 27 μL of Ultrapure water. Final concentration and purity was determined using the nanodrop 2000c and dsDNA concentration was quantified using Quan-iT Picogreen dsDNA reagent (Life Technologies, the Netherlands).

**Table 1:** Significant differentially abundant taxa between MS and CTR groups as calculated by Wilcoxon rank test at genus level, indicated by the *p*-value.

	Taxa	*p*	FDR	Ctrl	MS
Phylum	Bacteroidetes	0.012	0.16	0.68	0.54
	Spirochaetes	0.036	0.21	0.0017	0.0029
	Firmicutes	0.049	0.21	0.18	0.3
Class	Bacteroidia	0.012	0.25	0.68	0.54
	Spirochaetes	0.036	0.32	0.0017	0.0029
	Clostridia	0.045	0.32	0.16	0.27
Order	Bacteroidales	0.012	0.41	0.68	0.54
	Spirochaetales	0.031	0.47	0.0015	0.0029
	Clostridiales	0.045	0.47	0.16	0.27
Family	Spirochaetaceae	0.031	0.56	0.0015	0.0029
	Ruminococcaceae	0.037	0.56	0.088	0.15
	Clostridiales	0.046	0.56	0.035	0.075
Genus	Oscillospira	0.014	0.6	0.033	0.093
	Paraprevotellaceae	0.021	0.6	0.0005	0.00017
	Lachnospiraceae	0.03	0.6	0.0062	0.012
	Treponema	0.031	0.6	0.0015	0.0029
	Desulfovibrionaceae	0.031	0.6	0.0022	0.0035
	Allobaculum	0.043	0.6	0.00083	0.0012
	Phascolarctobacterium	0.044	0.6	0.017	0.01
	Clostridiales	0.046	0.6	0.035	0.075

*Values for the two groups are medians of the relative abundance of the indicated genus (% of all sequences). The FDR *q*-values are adjusted *p*-values that correct for multiple testing at a defined false discovery rate (Benjamini and Hochberg, 1995)*.

Prior to sequencing, a total of 96 samples were pooled at equimolar concentrations resulting in a total of 2 μg dsDNA of 40 ng μL^-1^. The amplicon pool was send to Genewiz, United Kingdom and sequencing was carried out on an Illumina MiSeq instrument 2 bp × 300 bp. Illumina Mi-Seq raw data were paired, demultiplexed and processed using the Quantitative Insights Into Microbial Ecology toolkit (Caporaso et al., 2010b). In brief, 16S rRNA bacterial partial sequences were quality trimmed using the default parameters in QIIME and reads were then binned into operational taxonomic units (OTUs) at 97% sequence identity using open-reference OTU picking method in QIIME. A representative sequence for each phylotype was aligned against the Greengenes corset (DeSantis et al., 2006) using PyNAST (Caporaso et al., 2010a), with sequences classified using the Greengenes taxonomy via blast. The alignment was filtered to remove common gaps and a phylogenetic tree was constructed using FastTree (Price et al., 2009). For all OTU-based analyses, the original OTU table was rarified to a depth of 6000 sequences per sample (the fewest in a single sample) to minimize effects of sampling effort on the analysis. The Quantitative Insights Into Microbial Ecology toolkit was also used to generate weighted/unweighted UniFrac distance matrices (Lozupone et al., 2006) and alfa-diversity metrics, including OTU richness (unique OTUs), ChaoI richness estimation, and Faith’s phylogenetic diversity indices. All data are presented as mean ± SEM. The microbiome composition was analyzed using the Wilcoxon rank test using the statistical software package SPSS 21.0 (IBM). The rank test-Kruskal–Wallis test was used for comparison of abundant taxa. The statistical significance was indicated as follows: ^∗^*p* < 0.05; ^∗∗^*p* < 0.01; and ^∗∗∗^*p* < 0.001.

Random forest identifies the subset of most relevant features by constructing a collection of decision trees. Constructing trees incorporating only a random subset of the features, which in turn avoids overfitting, control variance. The random forest package for R (v4.6-7) was used with default settings and baseline error was calculated as previously described (Yatsunenko et al., 2012).

## Results

### Early Life Stress and 5-HTT Genotype Affect the Relative Abundance of Gut Specific Microbial Taxa

In this study, we investigated the effects of (i) MS (MS and CTR groups), (ii) 5-HTT genotype (5-HTT^+/+^, 5-HTT^+/-^, and 5-HTT^-/-^ groups), and (iii) the combination of MS and 5-HTT genotype (5-HTT^+/+^-, 5-HTT^+/-^-, 5-HTT^-/-^-MS, and -CTR groups) in male and female rats at PND 21. As mentioned in Section “Materials and Methods,” since short handled rats (15 min) are different from rats raised in standard animal facility rearing conditions, we chose to use the short handling protocol (15 min) to control for the effect of maternal care upon reunion with the mothers.

#### Exposure to Maternal Separation Causes a Shift in the Bacteroidetes:Firmicutes Ratio in Young Rats

The microbiota composition of the fecal samples collected from the MS rats showed a shift in the ratio between the two main bacterial phyla relative to the control group and was characterized by a decrease in *Bacteroidetes* (*p* = 0.012) and increase in Firmicutes (*p* = 0.049; **Table 1**). Within the Firmicutes, Ruminococcaceae (*p* = 0.037) and Clostridiales (*p* = 0.046) were over-represented in the MS rats compared to the control group. Notably, these differences observed in the gut microbiome composition were not sex-dependent as the analysis showed no significant effect of sex (*p* = 0.346). Next, we identified the altered relative abundance of microbial genera that was unique to either exposure to MS or an alteration in the 5-HTT expression. Interestingly, changes in Trapanoma, Allobaculum, Phascolarctobacterium, Paraprevotellaceae, and Clostridiales were detected only when comparing MS groups to CTR groups and not when comparing the 5-HTT genotypes (see 5-HTT Genotype Has Stronger Effects on Microbial Dysbiosis than Maternal Separation for details, **Table 1**).

#### 5-HTT Genotype Has Stronger Effects on Microbial Dysbiosis than Maternal Separation

Diminished expression of 5-HTT resulted in stronger disruption of the microbiome composition when compared to the effect of MS. This is supported by pronounced changes in the relative abundance of bacterial taxa in 5-HTT^-/-^ in comparison with 5-HTT^+/-^ and 5-HTT^+/+^ rats (**Table 2**). The relative abundance of *Fusobacterium* (*p* = 0.0034), Deferribacteres (*p* = 0.024), and Proteobacteria (*p* = 0.035) were significantly higher in 5-HTT^-/-^ rats compared to 5-HTT^+/+^ and 5-HTT^+/-^. Moreover, the Firmicutes phylum was significantly (*p* = 0.033) higher in both 5-HTT^+/-^ and 5-HTT^-/-^ rats compared to the 5-HTT^+/+^ group. On the family level, Desulfovibrionaceae (*p* = 0.00053), Fusobacteriaceae (*p* = 0.0034), and Deferribacteraceae (*p* = 0.024) were all significantly over-represented in the 5-HTT^-/-^ rats compared to 5-HTT^+/-^ and 5-HTT^+/+^ rats (see **Table 2** for complete list). Only Lachnospiraceae was significantly higher in both 5-HTT^-/-^ and 5-HTT^+/-^ rats compared to the wild type group (*p* = 0.02). Several microbial genera were altered in the 5-HTT genotype compared to the control. These genera which include *Prevotella*, Lachnospiraceae; Other, Lachnospira, Desulfovibrionaceae; Other, Bacteroidales; Other; Other, *Fusobacterium*, Clostridium, *Ruminococcus*, Gemella, *Mucispirillum*, *Desulfovibrio*, Ruminococcaceae; Other, Blautia, were altered only when comparing the 5-HTT genotype groups and not when comparing the MS to the CTR groups (**Table 2**). Collectively, the results confirm the drastic effect of alteration in the expression of 5-HTT on the microbial community and population levels.

**Table 2:** Significant differentially abundant taxa between 5-HTT^+/+^, 5-HTT^+/-^, and 5-HTT^-/-^ CTR groups as calculated by Wilcoxon rank test at genus level, indicated by the *p*-value.

	Taxa	*p*-value	FDR	5-HTT^+/+^	5-HTT^+/^^-^	5-HTT^-^^/^^-^
Phylum	*Fusobacterium*	0.0034	0.044	0.00033	0.00058	0.0015***
	Cyanobacteria	0.021	0.091	0.0025	0.0055*	0.0032
	Deferribacteres	0.024	0.091	0.00067	0.00092	0.0022*
	Firmicutes	0.033	0.091	0.18	0.3*	0.34*
	Proteobacteria	0.035	0.091	0.023	0.02	0.034*
Class	Deltaproteobacteria	0.00053	0.012	0.0032	0.0048	0.014***
	Fusobacteriia	0.0034	0.039	0.00033	0.00058	0.0015**
	Cyanobacteria;c__4C0d-2	0.021	0.14	0.0025	0.0055*	0.0032
	Deferribacteres	0.024	0.14	0.00067	0.00092	0.0022*
	Clostridia	0.046	0.21	0.16	0.28	0.31*
Order	Desulfovibrionales	0.00053	0.019	0.0032	0.0048	0.014***
	Fusobacteriales	0.0034	0.06	0.00033	0.00058	0.0015**
	Cyanobacteria;c__4C0d-2;o__YS2	0.021	0.21	0.0025	0.0055*	0.0032
	Deferribacterales	0.024	0.21	0.00067	0.00092	0.0022*
	Clostridiales	0.046	0.27	0.16	0.28	0.31*
	Gemellales	0.046	0.27	0	0.00017*	0.000085*
Family	Desulfovibrionaceae	0.00053	0.033	0.0032	0.0048	0.014***
	Fusobacteriaceae	0.0034	0.11	0.00033	0.00058	0.0015**
	Bacteroidales;Other	0.02	0.23	0.0013	0.0032*	0.0025
	Lachnospiraceae	0.02	0.23	0.0095	0.022*	0.017*
	Cyanobacteria;c__4C0d-2;o__YS2;f__	0.021	0.23	0.0025	0.0055*	0.0032*
	Deferribacteraceae	0.024	0.23	0.00067	0.00092	0.0022*
	Prevotellaceae	0.026	0.23	0.038	0.027	0.014*
	Ruminococcaceae	0.031	0.24	0.088	0.14*	0.18*
	Gemellaceae	0.046	0.32	0	0.00017*	0.000085*
Genus	Desulfovibrionaceae;g__	0.00086	0.068	0.0022	0.0032	0.0094***
	*Desulfovibrio*	0.0011	0.068	0.00067	0.0012***	0.0033***
	*Fusobacterium*	0.0034	0.14	0.00033	0.00058	0.0015**
	Ruminococcus	0.017	0.26	0.004	0.0072*	0.0046
	Ruminococcaceae;Other	0.018	0.26	0.0005	0.001*	0.001*
	Oscillospira	0.019	0.26	0.033	0.065	0.11*
	Bacteroidales;Other;Other	0.02	0.26	0.0013	0.0032*	0.0025*
	Cyanobacteria;c__4C0d-2;o__YS2;f__;g__	0.021	0.26	0.0025	0.0055*	0.0032
	Ruminococcus	0.022	0.26	0.001	0.0022*	0.001
	*Mucispirillum*	0.024	0.26	0.00067	0.00092	0.0022*
	Lachnospiraceae;g__	0.026	0.26	0.0062	0.013*	0.0098
	Prevotella	0.027	0.26	0.038	0.027	0.014*
	Clostridium	0.029	0.26	0.00033	0.00084*	0.00042
	Lachnospiraceae;Other	0.029	0.26	0.00033	0.00058*	0.00067*
	Blautia	0.042	0.31	0.00033	0.0012*	0.00067
	Gemella	0.046	0.31	0	0.00017*	0.000085*
	Desulfovibrionaceae;Other	0.048	0.31	0	0	0^∗^
	Anaerovorax	0.048	0.31	0	0	0^∗^
	Lachnospira	0.048	0.31	0	0	0^∗^

*Values for the three groups are medians of the relative abundance of the indicated genus (% of all sequences). The FDR *q*-values are adjusted *p*-values that correct for multiple testing at a defined false discovery rate (Benjamini and Hochberg, 1995). The value “0” refers to a value < 0.00001. ^∗^*p* < 0.05, ^∗∗^*p* < 0.01, ^∗∗∗^*p* < 0.001*.

### Rats with Diminished 5-HTT Expression Are Associated with Inflammatory- and Depression-Type Microbiota When Exposed to Maternal Separation

**FIGURE 1:**
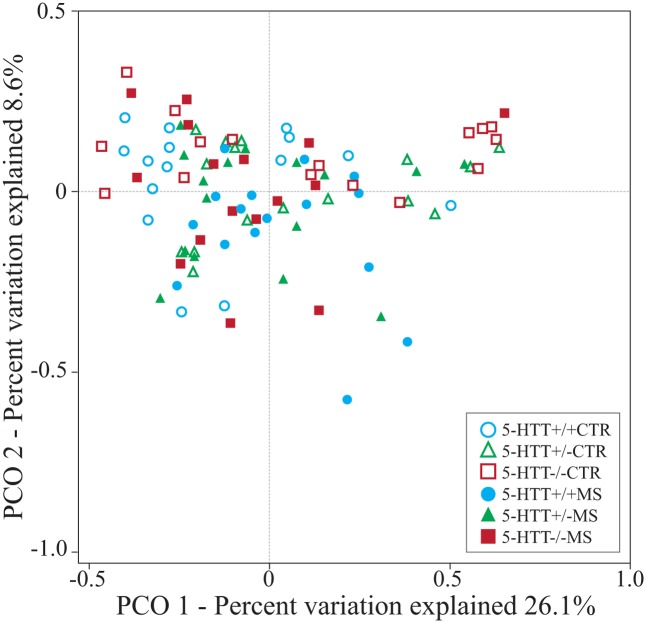
Principal coordinate analysis (PCO) based on weighted Unifrac distances between samples. The biplot displays the unconstrained distribution of the data. Statistical support for differences between sample types and across treatments was obtained by PEMANOVA using 10^3^ permutations.

Hierarchical clustering using PCO analysis based on weighted Unifrac distances revealed a significant effect of the exposure to MS in young rats with altered expression of 5-HTT (pseudo-*F* = 1.66, *p* = 0.008) (**Figure 1**). Overall, samples roughly segregated according to 5-HTT^+/+^ (*n* = 31), 5-HTT^+/-^ (overlap with 5-HTT^+/+^) (*n* = 32) and 5-HTT^-/-^ (*n* = 32) in the first axis *x* (26.1% of variation explained) and between control treatment and MS, in the second axis *y* (8.6% of variation explained). Interestingly, as expected, the distribution of the HTT^+/-^ samples is clustered at intermediate locations between the 5-HTT^+/+^ and 5-HTT^-/-^ samples. Moreover, the CTR 5-HTT^+/+^, 5-HTT^+/-^, and 5-HTT^-/-^ rats showed a tendency to lower richness in microbiome composition (*p* = 0.07) compared to the MS groups as measured by Faith’s phylogenetic diversity (PD).

**FIGURE 2:**
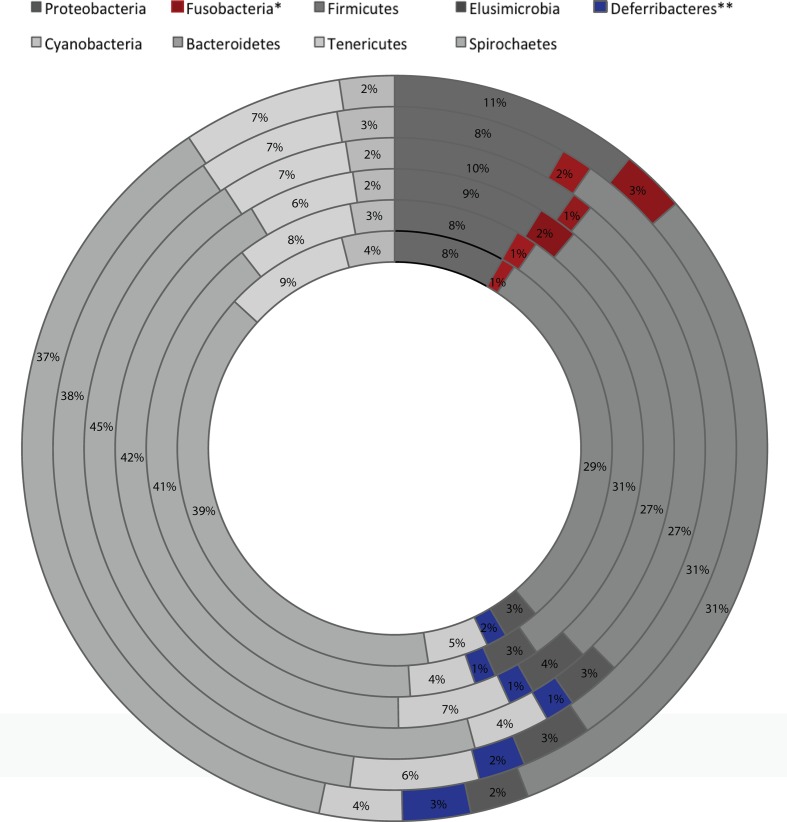
Global average microbial composition of fecal 3 weeks old rats samples (*n* = 8 per group) at phylum-level. ^∗^, ^∗∗^ indicate bacterial group significantly different among the six groups (from innermost to outermost circles; 5-HTT^+/+^-MS, 5-HTT^+/-^-MS, 5-HTT^-/-^-MS, 5-HTT^+/+^-CTR, 5-HTT^+/-^-CTR, 5-HTT^-/-^-CTR).

Analysis of the taxonomic composition at the highest assigned taxonomic level in the different 5-HTT genotype groups exposed to early life stress showed significant differences in the relative abundance of several bacterial groups. At the phylum level, significant changes in the relative abundance of Fusobacteria and Differbacteres (**Figure 2**) were detected. That is, Differebacteres and Fusobacteria were significantly more abundant in the 5-HTT^-/-^ CTR and MS groups compared to the 5-HTT^+/+^ and 5-HTT^+/-^ groups (*p* = 0.025) and *Fusobacterium* was even significantly higher in the 5-HTT^-/-^ CTR group compared to the 5-HTT^-/-^ MS group (*p* = 0.0065). These results indicate a significant effect of complete knockout of 5-HTT. There were seven statistically significant differences detected in the microbial composition at the family level as shown in **Table 3**. Prevotellaceae (*p* = 0.02), was decreased in the 5-HTT^-/-^ groups compared to 5-HTT^+/+^ and 5-HTT^+/-^ groups, whereas Fusobacteriaceae (*p* = 0.0029) and Gemellaceae (*p* = 0.015) were increased in the 5-HTT^+/-^ and 5-HTT^-/-^ groups, and Desulfovibrionaceae (*p* = 0.0045) were increased in the 5-HTT^-/-^ groups compared to the 5-HTT^+/+^ and 5-HTT^+/-^ groups. Only two bacterial families appeared to be influenced by the MS as well as the complete knockout of 5-HTT; Deferribacteraceae (*p* = 0.027), and Bacteroidales (*p* = 0.0084). The former was highest in 5-HTT^-/-^ CTR group and the latter was highest in the 5-HTT^-/-^ MS group (**Table 3**).

**Table 3:** Significant differentially abundant taxa between 5-HTT^+/-^, 5-HTT^-/-^, and 5-HTT^+/+^ -MS and CTR groups as calculated by Wilcoxon rank test at genus level, indicated by the *p*-value.

	Taxa	*p*-value	FDR	5-HTT-/- CTR	5-HTT-/- MS	5-HTT-/- CTR	5-HTT+/- MS	5-HTT+/+ CTR	5-HTT+/+ MS
Class	Fusobacteriia	0.0029	0.056	0.0015**	0.00066	0.00058	0.00067	0.00033	0.00042
	Deltaproteobacteria	0.0045	0.056	0.014**	0.005	0.0048	0.0038	0.0032	0.0039
	Deferribacteres	0.027	0.22	0.0022*	0.00058	0.00092	0.0005	0.00067	0.0005
Order	Fusobacteriales	0.0029	0.085	0.0015**	0.00066	0.00058	0.00067	0.00033	0.00042
	Desulfovibrionales	0.0045	0.085	0.014**	0.005	0.0048	0.0038	0.0032	0.0039
	Gemellales	0.015	0.19	0.000085	0.00017	0.00017	0	0	0
	Deferribacterales	0.027	0.26	0.0022*	0.00058	0.00092	0.0005	0.00067	0.0005
Family	Fusobacteriaceae	0.0029	0.16	0.0015**	0.00066	0.00058	0.00067	0.00033	0.00042
	Desulfovibrionaceae	0.0045	0.16	0.014**	0.005	0.0048	0.0038	0.0032	0.0039
	Bacteroidales;f__	0.0084	0.19	0.04*	0.057**	0.04*	0.045*	0.026	0.033
	Gemellaceae	0.015	0.26	0.000085*	0.00017*	0.00017*	0	0	0
	Prevotellaceae	0.02	0.28	0.014	0.038	0.027	0.033	0.038	0.039
	Deferribacteraceae	0.027	0.31	0.0022*	0.00058	0.00092	0.0005	0.00067	0.0005
	Bacteroidales;Other	0.046	0.45	0.0025*	0.0032*	0.0032*	0.0023*	0.0013	0.0018
Genus	*Fusobacterium*	0.0029	0.22	0.0015**	0.00066	0.00058	0.00067	0.00033	0.00042
	Desulfovibrionaceae;g__	0.0032	0.22	0.0094**	0.0035	0.0032	0.0026	0.0022	0.0035
	Bacteroidales;f__;g__	0.0084	0.32	0.04*	0.057**	0.04*	0.045*	0.026	0.033
	*Desulfovibrio*	0.0094	0.32	0.0033**	0.0014	0.0012	0.0013	0.00067	0.00075
	Gemella	0.015	0.34	0.000085*	0.00017*	0.00017*	0	0	0
	Ruminococcus	0.015	0.34	0.0046	0.0042	0.0072*	0.0077*	0.004	0.0063*
	Phascolarctobacterium	0.017	0.34	0.02*	0.014	0.015	0.0084	0.017	0.01
	Prevotella	0.02	0.34	0.014*	0.038	0.027	0.033	0.038	0.039
	*Mucispirillum*	0.027	0.37	0.0022*	0.00058	0.00092	0.0005	0.00067	0.0005
	Clostridium	0.03	0.37	0.00042	0.00042	0.00084*	0.00067*	0.00033	0.00084*
	Roseburia	0.04	0.4	0.00033	0^∗^	0.00025	0.000085	0.00017	0.00025
	Ruminococcaceae;Other	0.043	0.4	0.001*	0.0005	0.001*	0.0005	0.0005	0.00075
	Oscillospira	0.045	0.4	0.11*	0.062	0.065	0.09*	0.033	0.093*
	Bacteroidales;Other;Other	0.046	0.4	0.0025*	0.0032*	0.0032*	0.0023*	0.0013	0.0018

*Values for the six groups are medians of the relative abundance of the indicated genus (% of all sequences). The FDR *q*-values are adjusted *p*-values that correct for multiple testing at a defined false discovery rate (Benjamini and Hochberg, 1995). The value “0” refers to a value < 0.00001. ^∗^*p* < 0.05, ^∗∗^*p* < 0.01, ^∗∗∗^*p* < 0.001*.

**FIGURE 3:**
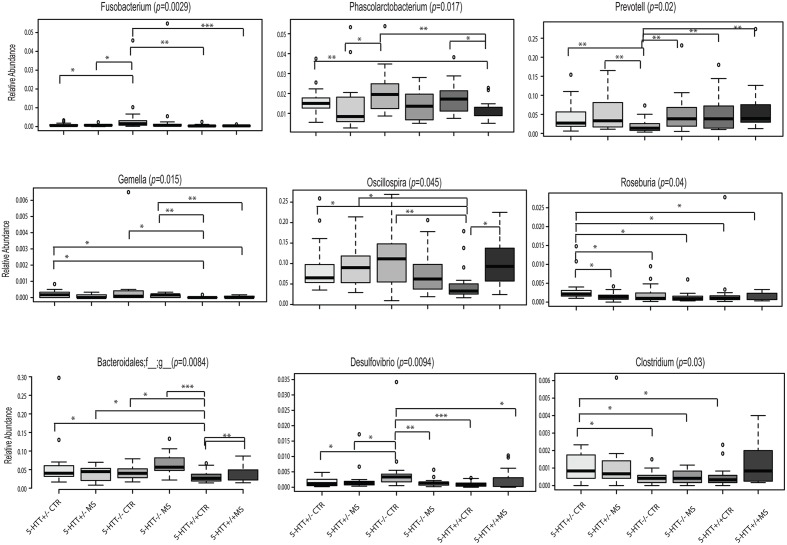
Altered Gut Microbiota Composition. Significant differences of selected bacteria at genus level among the six groups. ^∗^*p* < 0.05, ^∗∗^*p* < 0.01, ^∗∗∗^*p* < 0.001.

At the genus level, *Fusobacterium* (*p* = 0.029), *Oscillospira* (*p* = 0.045), *Mucispirillum* (*p* = 0.027), and *Desulfuvibrio* (*p* = 0.0094) were over-represented in the 5-HTT^-/-^ CTR group, whereas genera of the family Bacteroidales (*p* = 0.0084) were increased in the 5-HTT^-/-^ MS group. In contrast, *Prevotella* (*p* = 0.02) was under-represented in the 5-HTT^-/-^ CTR group and Roseburia (*p* = 0.04) was over represented in the 5-HTT^+/-^ CTR group (**Figure 3** and **Table 3**).

Several OTUs were altered in abundance in maternally separated, 5-HTT^+/-^, 5-HTT^-/-^ rats. The complete list of OTUs and respective sequences that differed significantly between the six groups of rats is shown in Supplementary Table 1. Collectively, these results indicate that diminished expression of 5-HTT induces dysbiosis in young animals, which could persist into adulthood.

### Early Life Stress- and 5-HTT Genotype Subgroups Identification Based on Microbiome Signatures

The differences in the gut microbiota composition from the 16S rDNA data between the six groups were assessed by ordination (Supplementary Figure 2). Statistics based on random permutations of the redundancy analysis (RDA) showed that the MS groups could significantly be separated at genus level (*p* < 0.001) from the CTR groups. The centroids of the 5-HTT^+/+^-, 5-HTT^+/-^, and 5-HTT^-/-^- CTR groups were clearly separated; indicating a strong effect of 5-HTT genotype on the microbiota composition. As expected, the 5-HTT^+/-^ -CTR group was in an intermediate position between the 5-HTT^+/+^- and 5-HTT^-/-^- CTR groups. Interestingly, the 5-HTT^+/+^ and 5-HTT^+/-^- MS groups were clustered together and very close to the 5-HTT^-/-^-MS group. The results point to a different effect of exposure to early-life MS on the microbiota composition when compared to the 5-HTT^+/+^-, 5-HTT^+/-^-, and 5-HTT^-/-^- CTR groups, irrespective of the genetic background (5-HTT genotype). The RDA results support the results obtained from the PCO analysis of the taxonomic composition at the OTU level (Supplementary Table 1).

Moreover, random forest analysis provided further support for the differentiation of early life stress- and 5-HTT genotype subgroups. OTUs contributing to the differentiation of the gut microbial communities among the groups, according to their random forest importance (mean decrease in accuracy scores), included members of the genera *Paraprevotella*, *Prevotella*, *Sutterella*, *Oscillospira*, and *Bacteroides* (Supplementary Table 2). As such, these patterns in microbiota shifts point to a signature that discriminates between the genotypes relating to altered levels of gene expression of the 5-HTT and between the groups with and without early life stress.

## Discussion

The adverse early life conditions have been linked to increased sensitivity to anxiety- and depression-like behavior in subjects with lower expression of 5-HTT (Carola et al., 2008; van den Hove et al., 2011). However, to the best of our knowledge, the effect of the combination of these factors on the microbiota composition has yet not been investigated. Our study shows that the 5-HTT genotype, especially when combined with early life stress results in a state of microbiota dysbiosis. This dysbiosis is characterized by abundance distribution of members of the gut microbiota previously reported to be associated with a state of intestinal inflammation, including inflammation typically seen in cases of brain disorders such as autism, major depressive disorder and Parkinson’s disease. This is intriguing taking into account the role of 5-HTT in the gut. 5-HTT plays a key role in clearance of 5-HT by its rapid uptake to maintain optimal extracellular availability of 5-HT in the gut to facilitate its physiological actions and prevent receptor desensitization (Gill et al., 2013) In fact, several lines of evidence support downregulation of 5-HTT in inflammatory (Martel, 2006) or diarrheal disorders (Foley et al., 2007). In addition, recent studies have demonstrated 5-HTT downregulation via alterations in its gene expression in response to pro-inflammatory agents such as TNF and IFN-g. We and others have previously shown that opportunistic commensals (also known as pathobionts) flourish in an inflammatory gut environment [which is known to be associated with early life stress (Lennon et al., 2013) and 5-HTT genotype (Bischoff et al., 2009; Haub et al., 2010)] resulting in an imbalance in the microbiota community (Gaboriau-Routhiau et al., 2009; El Aidy et al., 2014). Thus, our data refer to a causal effect of 5-HTT genotype and/or early stress in the observed state of microbial dysbiosis.

We detected an increase in the abundance of Desulfovibrionaceae in the 5-HTT^-/-^ groups compared to the 5-HTT^+/+^ and 5-HTT^+/-^ groups. The over-representation of *Desulfovibrio* in the gut causes increased production of sulfide from sulfate contained in diet, which can lead to structural and functional changes in the gut barrier as well as the gut associated immune system. Hydrogen sulfide is toxic to the gut as well as the mucosal gene expression, thus resulting in inflammation. Moreover, *Desulfovibrio* competes with butyrate-producing bacteria for the lactate leading to production of higher amounts of propionic acid, which have been shown to produce autism-like manifestations in animals (Macfabe, 2012). The 5-HTT^-/-^ phenotype was also associated with abundance in *Mucispirillum*, which belongs to the family Deferribacteraceae and inhabits the mucus layer in the colon. *Mucispirillum* has been associated with intestinal inflammation (El Aidy et al., 2014; Rooks et al., 2014). Of note, *Mucispirillum* and *Desulfovibrio* are among the colitogenic gut microbiomes, being used as microbial markers in active colitis owing to their opportunistic nature given the putative capacity of *Mucispirillum* to degrade mucin (Robertson et al., 2005; Berry et al., 2012) and *Desulfovibrio* to produce high levels of hydrogen sulfide during active inflammation (Carbonero et al., 2012), which may further fuel inflammation. Similarly, *Fusobacterium* was found in higher amounts in patients with inflammatory bowel syndrome and even reductions in the titer of antibody to particular strains of *Fusobacterium* appeared to correlate to improved inflammation (DiBaise et al., 2012).

Among the microbiome signature identified in this study to distinguish between early life stress- and 5-HTT genotype subgroups (Supplementary Table 2) are *Sutterella, Prevotella. Sutterella* has been reported to be associated with gastrointestinal infections in humans (Engberg et al., 2000) and to be highly prevalent in biopsies taken from the gut of autistic children with gastrointestinal disturbance compared to controls (Wang et al., 2013). Members of the Prevotellaceae family and subsequently in the *Paraprevotella* and *Prevotella* genera, were found to be at low relative abundance in patients with PD patients (Gaboriau-Routhiau et al., 2011; Scheperjans et al., 2014) as well as in depressed patients (Dinan, 2009). In summary, our results closely align to previous reports showing a link between altered microbiome, inflammatory disorders and several neuronal disorders.

A limitation in this study is the lack of an additional control group with rats that were raised under standard animal facility rearing conditions. Psychological stress can influence the gut microbiome composition, and short handling is considered to be a psychological stressor. The microbiome composition might therefore have been influenced by the short handling procedure used in the present study. Recently Allen-Blevins et al. (2017) showed that the fecal composition in handled animals was significant altered compared with non-handled animals. A significant decrease in *Bifidobacterium, Bacteroides*, and *Bacteroidetes* was found in the gut microbiota of handled mice compared with non-handled mice. Future studies need to be executed to investigate what influence the short handling procedure in the present study has on the gut microbiome composition compared with standard animal facility rearing rats.

## Conclusion

We demonstrate that lower 5-HTT expression levels and early-life stress induced disruptions in the gut microbes of young rats, with no differences between males and females. Analysis of the gut microbiota of the different 5-HTT genotypes combined with MS showed altered microbial composition with abundance of members previously shown to be associated with intestinal inflammatory disorders and disturbed gut barrier, which are co-morbidities of several neuronal disorders. These results indicate that exposure to transient early-life adverse effects in young rats with altered expression of the 5-HTT has effects on the gut microbiota on PND21. MS by itself also showed a shift in the ratio between the two main bacterial phyla, irrespective of the 5-HTT genotype. Interestingly, the effect of the 5-HTT genotype on microbiota dysbiosis was more pronounced than the effect of MS, indicating an important role of 5-HT signaling during development. New experiments are needed to explore the functional networks of microbes that are altered as a result of 5-HTT genotype and/or early life stress. Naturally, these networks might change over time and it is worth investigating how they would affect the gut-brain axis. Thus, prospective experiments are necessary to examine possible long-term effects of early-life stress on gut microbiota in 5-HTT^+/+^, 5-HTT^+/-^, and 5-HTT^-/-^ rats and link this to affective and social behavior during adulthood. Overall, our data hint into the direction that the absence as well as the exacerbation of certain bacterial taxa in the gut of early-life stressed rats may represent risk factors for the development of depression, neurodegenerative disorders and inflammatory diseases such as IBS. Restoring members of the microbiota with neuro-immune-regulatory functions may prevent an overly robust stress-induced inflammatory response, which may contribute to the onset of mental illnesses.

## Author Contributions

Drafting and/or revising the paper, including final approval to publish: SE, AR, FD-A, RE, DH, JS, JO.

## Conflict of Interest Statement

The authors declare that the research was conducted in the absence of any commercial or financial relationships that could be construed as a potential conflict of interest.
