# Supplementary material for: Serotonin Transporter Genotype Modulates the Gut Microbiota Composition in Young Rats, an Effect Augmented by Early Life Stress
Source: Front Cell Neurosci. 2017 Aug 3;11:222. doi: 10.3389/fncel.2017.00222 (PMC5540888; doi:10.3389/fncel.2017.00222)
Supplement: Supplementary file 1 [file Data_Sheet_1.DOCX]

**Supplemental Figure 1.** Timeline of the experiment. Female heterozygous serotonin transporter knockout rats (5-HTT^+/−^) were crossed with 5-HTT^+/−^ males resulting in offspring of all genotypes (5-HTT^+/+^, 5-HTT^+/−^ and 5-HTT^−/−^). Day of birth was designated as postnatal day (PND) 0. Litters were randomly allocated to one of two rearing conditions (from PND2 to PND15): maternal separation for 360 min or control handling for 15 min. Fecal samples were collected when male and female 5-HTT^+/+^, 5-HTT^+/−^ and 5-HTT^−/−^ rats were 21 days old (PND21). After fecal collection, rats were weaned.

**Supplemental Figure 2.** Redundancy analysis (RDA) based on the OTU level showing a significant separation between the MS and CTL groups (*p*=0.001). The ellipses identify the centroids of each dataset.

**Supplemental Table 1**

Significant differentially abundant taxa between MS and CTR groups as calculated by Wilcoxon rank test at the OTU level, indicated by the p-value. Values for the six groups are medians of the relative abundance of the indicated genus (% of all sequences). The FDR q-values are adjusted p-values that correct for multiple testing at a defined false discovery rate (Benjamini et al., 1995)

| Taxa | P | FDR | 5-HTT^+/-^  CTR | 5-HTT^+/-^  MS | 5-HTT^-/-^  CTR | 5-HTT^-/-^  MS | 5-HTT^+/+^  CTR | 5-HTT^+/+^  MS |
| --- | --- | --- | --- | --- | --- | --- | --- | --- |
| k__Bacteria__p__Firmicutes__c__Clostridia__o__Clostridiales__f____g____  s___19593 | 0.00044 | 0.09 | 2 | 4.5 | 1 | 2 | 0 | 6.5 |
| k__Bacteria__p__Proteobacteria__c__Deltaproteobacteria__  o__Desulfovibrionales__f__Desulfovibrionaceae__g____s___New.ReferenceOTU5242 | 0.0006 | 0.09 | 1.5 | 2.5 | 7.5 | 2 | 2 | 1 |
| k__Bacteria__p__Bacteroidetes__c__Bacteroidia__o__Bacteroidales__  f__S24.7__g____s___278675 | 0.0027 | 0.23 | 1 | 2 | 1 | 1 | 6 | 2.5 |
| k__Bacteria__p__Bacteroidetes__c__Bacteroidia__o__Bacteroidales__  f__S24.7__g____s___New.ReferenceOTU1632 | 0.004 | 0.23 | 12.5 | 36 | 12.5 | 17.5 | 20 | 27.5 |
| k__Bacteria__p__Bacteroidetes__c__Bacteroidia__o__Bacteroidales__  f__S24.7__g____s___New.ReferenceOTU2373 | 0.0053 | 0.23 | 4 | 5 | 2 | 4.5 | 7 | 6.5 |
| k__Bacteria__p__Firmicutes__c__Clostridia__o__Clostridiales__f  __Clostridiaceae__g____s___New.ReferenceOTU114 | 0.0067 | 0.23 | 3 | 2.5 | 1.5 | 0 | 2 | 1 |
| k__Bacteria__p__Bacteroidetes__c__Bacteroidia__o__Bacteroidales__  f__Bacteroidaceae__g__Bacteroides__s__eggerthii_316761 | 0.0072 | 0.23 | 3.5 | 2 | 3 | 4.5 | 5 | 1 |
| k__Bacteria__p__Firmicutes__c__Clostridia__o__Clostridiales__  f____g____s___400599 | 0.0074 | 0.23 | 3.5 | 3.5 | 7.5 | 1 | 3 | 2 |
| k__Bacteria__p__Bacteroidetes__c__Bacteroidia__o__Bacteroidales__  f__.Odoribacteraceae.__g__Butyricimonas__s___New.ReferenceOTU2797 | 0.0088 | 0.23 | 1.5 | 1.5 | 1 | 3 | 4 | 1.5 |
| k__Bacteria__p__Bacteroidetes__c__Bacteroidia__o__Bacteroidales__  f__.Paraprevotellaceae.__g__Paraprevotella__s___New.ReferenceOTU2809 | 0.0088 | 0.23 | 0 | 1 | 0 | 0 | 0 | 1 |
| k__Bacteria__p__Bacteroidetes__c__Bacteroidia__o__Bacteroidales__  f____g____s___1110064 | 0.01 | 0.23 | 10 | 8.5 | 5 | 17 | 7 | 7 |
| k__Bacteria__p__Firmicutes__c__Clostridia__o__Clostridiales__  f__Veillonellaceae__g__Phascolarctobacterium__s___916143 | 0.012 | 0.23 | 35 | 16.5 | 54.5 | 27 | 49 | 18 |
| k__Bacteria__p__Bacteroidetes__c__Bacteroidia__o__Bacteroidales__  f__.Odoribacteraceae.__g__Butyricimonas__s___360730 | 0.012 | 0.23 | 1 | 1.5 | 3 | 2.5 | 3 | 0 |
| k__Bacteria__p__Bacteroidetes__c__Bacteroidia__o__Bacteroidales__  f__S24.7__g____s___388276 | 0.013 | 0.23 | 5.5 | 5.5 | 1.5 | 3.5 | 5 | 5.5 |
| k__Bacteria__p__Bacteroidetes__c__Bacteroidia__o__Bacteroidales__  f__S24.7__g____s___97151 | 0.014 | 0.23 | 1 | 1.5 | 1 | 1 | 4 | 1 |
| k__Bacteria__p__Bacteroidetes__c__Bacteroidia__o__Bacteroidales__  f__S24.7__g____s___New.ReferenceOTU370 | 0.014 | 0.23 | 1 | 1.5 | 0 | 1 | 1 | 1 |
| k__Bacteria__p__Bacteroidetes__c__Bacteroidia__o__Bacteroidales__  f__Bacteroidaceae__g__Bacteroides__s___New.ReferenceOTU1806 | 0.014 | 0.23 | 0.5 | 4 | 0 | 1 | 1 | 0 |
| k__Bacteria__p__Bacteroidetes__c__Bacteroidia__o__Bacteroidales__  f__Bacteroidaceae__g__Bacteroides__s__uniformis_350277 | 0.014 | 0.23 | 3 | 1.5 | 3 | 4 | 1 | 0.5 |
| k__Bacteria__p__Bacteroidetes__c__Bacteroidia__o__Bacteroidales__  f__Bacteroidaceae__g__Bacteroides__s___3325758 | 0.015 | 0.24 | 0 | 0 | 0.5 | 1 | 0 | 0 |
| k__Bacteria__p__Bacteroidetes__c__Bacteroidia__o__Bacteroidales__  f__RF16__g____s___New.ReferenceOTU207 | 0.018 | 0.26 | 4.5 | 10.5 | 6 | 9.5 | 8 | 5.5 |
| k__Bacteria__p__Tenericutes__c__Mollicutes__o__RF39__  f____g____s___New.ReferenceOTU3836 | 0.018 | 0.26 | 1.5 | 0 | 0 | 0 | 0 | 1.5 |
| k__Bacteria__p__Elusimicrobia__c__Elusimicrobia__o__Elusimicrobiales__  f__Elusimicrobiaceae__g____s___New.ReferenceOTU5587 | 0.019 | 0.26 | 2.5 | 2.5 | 0.5 | 2 | 0 | 4 |
| k__Bacteria__p__Firmicutes__c__Clostridia__o__Clostridiales__  f____g____s___New.ReferenceOTU176 | 0.02 | 0.26 | 0.5 | 0 | 1 | 0 | 0 | 0 |
| k__Bacteria__p__Bacteroidetes__c__Bacteroidia__o__Bacteroidales__  f____g____s___New.ReferenceOTU1262 | 0.023 | 0.29 | 2 | 0.5 | 2.5 | 3 | 1 | 1 |
| k__Bacteria__p__Fusobacteria__c__Fusobacteriia__o__Fusobacteriales__  f__Fusobacteriaceae__g__Fusobacterium__s___809380 | 0.024 | 0.29 | 0 | 1 | 1 | 0 | 0 | 0 |
| k__Bacteria__p__Firmicutes__c__Clostridia__o__Clostridiales__  f__Ruminococcaceae__g__Oscillospira__s___407963 | 0.029 | 0.33 | 1 | 2 | 1 | 0 | 0 | 0 |
| k__Bacteria__p__Bacteroidetes__c__Bacteroidia__o__Bacteroidales__  f__Bacteroidaceae__g__Bacteroides__s__plebeius_New.ReferenceOTU2795 | 0.03 | 0.33 | 0 | 0 | 0 | 0 | 0 | 0 |
| k__Bacteria__p__Bacteroidetes__c__Bacteroidia__o__Bacteroidales__  f__.Paraprevotellaceae.__g__.Prevotella.__s___New.ReferenceOTU4583 | 0.035 | 0.37 | 0 | 0.5 | 2 | 0 | 1 | 1 |
| k__Bacteria__p__Bacteroidetes__c__Bacteroidia__o__Bacteroidales__  f____g____s___New.ReferenceOTU1593 | 0.036 | 0.37 | 2 | 4.5 | 1 | 2.5 | 2 | 3 |
| k__Bacteria__p__Bacteroidetes__c__Bacteroidia__o__Bacteroidales__  f__Bacteroidaceae__g__Bacteroides__s___New.ReferenceOTU5154 | 0.037 | 0.37 | 1 | 0 | 3 | 1 | 2 | 0.5 |
| k__Bacteria__p__Bacteroidetes__c__Bacteroidia__o__Bacteroidales__  f__.Paraprevotellaceae.__g__CF231__s___New.ReferenceOTU3668 | 0.039 | 0.38 | 0 | 0 | 0 | 0 | 0 | 0 |
| k__Bacteria__p__Bacteroidetes__c__Bacteroidia__o__Bacteroidales__  f__S24.7__g____s___New.ReferenceOTU1105 | 0.04 | 0.38 | 2 | 17 | 2.5 | 6.5 | 2 | 3.5 |
| k__Bacteria__p__Bacteroidetes__c__Bacteroidia__o__Bacteroidales__  f__Prevotellaceae__g__Prevotella__s__copri_New.ReferenceOTU1097 | 0.042 | 0.38 | 1 | 1.5 | 1 | 6 | 3 | 6.5 |
| k__Bacteria__p__Deferribacteres__c__Deferribacteres__o__Deferribacterales__  f__Deferribacteraceae__g__Mucispirillum__s__schaedleri_1871 | 0.044 | 0.39 | 1 | 1.5 | 4 | 1 | 1 | 0 |
| Unassigned_New.ReferenceOTU1629 | 0.046 | 0.39 | 0 | 0 | 0 | 0 | 0 | 0 |
| k__Bacteria__p__Bacteroidetes__c__Bacteroidia__o__Bacteroidales__  f__RF16__g____s___1110242 | 0.049 | 0.41 | 8 | 3.5 | 1.5 | 5 | 6 | 5 |

**Supplemental Table 2**

OTUs contributing to the differentiation of MS versus CTR 5-HTT gut communities

| **Taxa** | **Score (mean decrease accuracy)** |
| --- | --- |
| k__Bacteria__p__Bacteroidetes__c__Bacteroidia__o__Bacteroidales__f__Bacteroidaceae__g__Bacteroides__s___ | 0.08 |
| k__Bacteria__p__Bacteroidetes__c__Bacteroidia__o__Bacteroidales__f__.Paraprevotellaceae.__g__CF231__s___ | 0.11 |
| k__Bacteria__p__Firmicutes__c__Clostridia__o__Clostridiales__f__Ruminococcaceae__g____s___ | 0.31 |
| k__Bacteria__p__Tenericutes__c__Mollicutes__o__RF39__f____g____s___ | 0.32 |
| k__Bacteria__p__Bacteroidetes__c__Bacteroidia__o__Bacteroidales__f__Bacteroidaceae__g__Bacteroides__s___ | 0.38 |
| k__Bacteria__p__Bacteroidetes__c__Bacteroidia__o__Bacteroidales__f__S24.7__g____s___ | 0.44 |
| k__Bacteria__p__Bacteroidetes__c__Bacteroidia__o__Bacteroidales__f__S24.7__g____s___ | 0.52 |
| k__Bacteria__p__Proteobacteria__c__Betaproteobacteria__o__Burkholderiales__f__Alcaligenaceae__g__Sutterella__s___ | 0.63 |
| k__Bacteria__p__Bacteroidetes__c__Bacteroidia__o__Bacteroidales__f__S24.7__g____s___ | 0.65 |
| k__Bacteria__p__Bacteroidetes__c__Bacteroidia__o__Bacteroidales__f__.Paraprevotellaceae.__g__CF231__s  ___ | 0.75 |
| k__Bacteria__p__Proteobacteria__c__Betaproteobacteria__o__Burkholderiales__f__Alcaligenaceae__g__Sutterella__s___ | 0.81 |
| k__Bacteria__p__Bacteroidetes__c__Bacteroidia__o__Bacteroidales__f__Bacteroidaceae__g__Bacteroides__s___ | 0.81 |
| k__Bacteria__p__Bacteroidetes__c__Bacteroidia__o__Bacteroidales__f__.Paraprevotellaceae.__g__.Prevotella.__s___ | 0.89 |
| k__Bacteria__p__Firmicutes__c__Bacilli__o__Lactobacillales__f__Lactobacillaceae__g__Lactobacillus__s___ | 1.06 |
| k__Bacteria__p__Cyanobacteria__c__4C0d.2__o__YS2__f____g____s___ | 1.15 |
| k__Bacteria__p__Proteobacteria__c__Gammaproteobacteria__o__Enterobacteriales__f__Enterobacteriaceae__g____s___ | 1.26 |
| k__Bacteria__p__Bacteroidetes__c__Bacteroidia__o__Bacteroidales__f__Prevotellaceae__g__Prevotella__s___ | 1.52 |
| k__Bacteria__p__Cyanobacteria__c__4C0d.2__o__YS2__f____g____s___ | 1.56 |
| k__Bacteria__p__Bacteroidetes__c__Bacteroidia__o__Bacteroidales__f__.Paraprevotellaceae.__g__CF231__s___ | 1.91 |
| k__Bacteria__p__Bacteroidetes__c__Bacteroidia__o__Bacteroidales__f__S24.7__g____s___ | 2.1 |
| k__Bacteria__p__Firmicutes__c__Clostridia__o__Clostridiales__f____g____s___ | 2.25 |
| k__Bacteria__p__Bacteroidetes__c__Bacteroidia__o__Bacteroidales__f__Bacteroidaceae__g__Bacteroides__s___ | 2.48 |
| k__Bacteria__p__Bacteroidetes__c__Bacteroidia__o__Bacteroidales__f__Bacteroidaceae__g__Bacteroides__s___ | 2.53 |
| k__Bacteria__p__Bacteroidetes__c__Bacteroidia__o__Bacteroidales__f__.Paraprevotellaceae.__g__Paraprevotella__s___ | 2.74 |
| k__Bacteria__p__Bacteroidetes__c__Bacteroidia__o__Bacteroidales__f__RF16__g____s___ | 2.97 |
| k__Bacteria__p__Bacteroidetes__c__Bacteroidia__o__Bacteroidales__f__Prevotellaceae__g__Prevotella__s__copri_ | 3.2 |
| k__Bacteria__p__Bacteroidetes__c__Bacteroidia__o__Bacteroidales__f__S24.7__g____s___ | 3.4 |
| k__Bacteria__p__Bacteroidetes__c__Bacteroidia__o__Bacteroidales__f____g____s___ | 3.65 |
| k__Bacteria__p__Bacteroidetes__c__Bacteroidia__o__Bacteroidales__f__.Odoribacteraceae.__g__Butyricimonas__s___ | 3.8 |
| k__Bacteria__p__Bacteroidetes__c__Bacteroidia__o__Bacteroidales__f____g____s___ | 3.87 |
| k__Bacteria__p__Firmicutes__c__Clostridia__o__Clostridiales__f__Ruminococcaceae__g__Oscillospira__s___ | 3.88 |
| k__Bacteria__p__Bacteroidetes__c__Bacteroidia__o__Bacteroidales__f__S24.7__g____s___ | 3.89 |
| k__Bacteria__p__Bacteroidetes__c__Bacteroidia__o__Bacteroidales__f__Bacteroidaceae__g__Bacteroides__s___ | 4.12 |
| k__Bacteria__p__Bacteroidetes__c__Bacteroidia__o__Bacteroidales_ | 4.35 |
| k__Bacteria__p__Bacteroidetes__c__Bacteroidia__o__Bacteroidales__f__Bacteroidaceae__g__Bacteroides__s___ | 4.77 |
| k__Bacteria__p__Bacteroidetes__c__Bacteroidia__o__Bacteroidales__f__Prevotellaceae__g__Prevotella__s__copri_ | 4.88 |
| k__Bacteria__p__Bacteroidetes__c__Bacteroidia__o__Bacteroidales__f____g____s___ | 4.9 |
| k__Bacteria__p__Bacteroidetes__c__Bacteroidia__o__Bacteroidales__f__.Paraprevotellaceae.__g__YRC22__s___ | 5.09 |
| k__Bacteria__p__Firmicutes__c__Clostridia__o__Clostridiales__f__Ruminococcaceae__g__Oscillospira__s___ | 5.14 |
| k__Bacteria__p__Bacteroidetes__c__Bacteroidia__o__Bacteroidales__f__S24.7__g____s___ | 5.39 |
| k__Bacteria__p__Bacteroidetes__c__Bacteroidia__o__Bacteroidales__f__Bacteroidaceae__g__Bacteroides__s__uniformis_ | 5.69 |
| k__Bacteria__p__Bacteroidetes__c__Bacteroidia__o__Bacteroidales__f____g____s___ | 5.82 |
| k__Bacteria__p__Bacteroidetes__c__Bacteroidia__o__Bacteroidales__f__Bacteroidaceae__g__Bacteroides__s__eggerthii_ | 5.84 |
| k__Bacteria__p__Bacteroidetes__c__Bacteroidia__o__Bacteroidales__f__RF16__g____s___ | 5.87 |
| k__Bacteria__p__Firmicutes__c__Clostridia__o__Clostridiales__f____g____s___ | 6.14 |
| k__Bacteria__p__Bacteroidetes__c__Bacteroidia__o__Bacteroidales__f__S24.7__g____s___ | 7.7 |
| k__Bacteria__p__Bacteroidetes__c__Bacteroidia__o__Bacteroidales__f____g____s___ | 9.27 |
| k__Bacteria__p__Bacteroidetes__c__Bacteroidia__o__Bacteroidales__f__S24.7__g____s___ | 10.17 |
| k__Bacteria__p__Firmicutes__c__Clostridia__o__Clostridiales__f__Ruminococcaceae__g__Oscillospira__s___ | 10.61 |
| k__Bacteria__p__Bacteroidetes__c__Bacteroidia__o__Bacteroidales__f__S24.7__g____s___ | 10.69 |
| k__Bacteria__p__Bacteroidetes__c__Bacteroidia__o__Bacteroidales__f__Bacteroidaceae__g__Bacteroides__s___ | 11.21 |
| k__Bacteria__p__Firmicutes__c__Clostridia__o__Clostridiales__f__Veillonellaceae__g__Phascolarctobacterium__s___ | 13.17 |
| k__Bacteria__p__Bacteroidetes__c__Bacteroidia__o__Bacteroidales__f__S24.7__g____s___ | 14.25 |
| k__Bacteria__p__Firmicutes__c__Clostridia__o__Clostridiales__f____g____s___ | 16.12 |
