# Supplementary figures and images for: Serotonin Transporter Genotype Modulates the Gut Microbiota Composition in Young Rats, an Effect Augmented by Early Life Stress
Source: Front Cell Neurosci. 2017 Aug 3;11:222. doi: 10.3389/fncel.2017.00222 (PMC5540888; doi:10.3389/fncel.2017.00222)

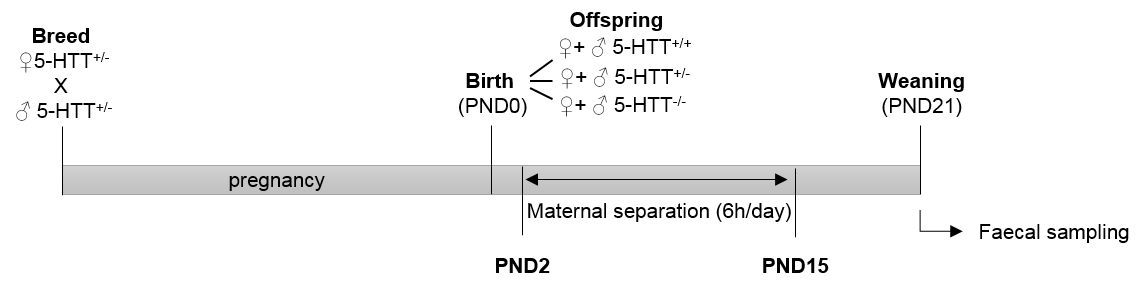

Supplement: Supplementary file 2 [file Image_1.TIF]

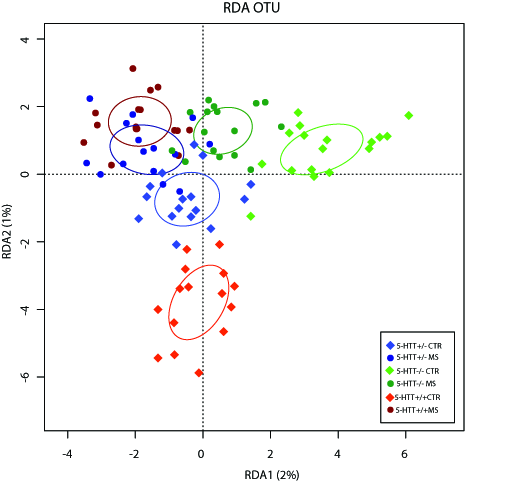

Supplement: Supplementary file 3 [file Image_2.TIF]
